# Association between serum sodium and sporadic Parkinson’s disease

**DOI:** 10.3389/fnins.2025.1555831

**Published:** 2025-03-25

**Authors:** Wen Zhou, Qingqing Xia, Duan Liu, Jun-ying Li, Liang Gong

**Affiliations:** Chengdu Second People’s Hospital, Chengdu, Sichuan, China

**Keywords:** sporadic Parkinson’s disease, serum sodium, retrospective cross-sectional study, Parkinson’s progression markers Initiative, pPMI

## Abstract

**Background:**

The correlation between serum sodium and sporadic Parkinson’s disease remains unclear currently. This study aimed to assess the association between serum sodium and sporadic Parkinson’s disease.

**Objective:**

The ultimate goal is to gain a deeper understanding of the implications of this relationship between serum sodium and sporadic Parkinson’s disease.

**Methods:**

We conducted a retrospective cross-sectional study involving 1,189 participants in PPMI cohort. Age, sex, education years, race, body mass index, calcium, alanine aminotransferase, aspartate aminotransferase, white blood cell, lymphocytes, neutrophils, monocytes, red blood cell, hemoglobin, platelets, total protein, albumin, serum uric acid, serum sodium, serum potassium, urea nitrogen, creatinine, serum glucose were obtained from all participants. Logistic regression, and smooth curve fitting were utilized to substantiate the research objectives.

**Results:**

The overall sporadic Parkinson’s disease was 77.5% (921/1189); it was 71.9% (143/199), 75.4% (295/391), 76.7% (171/223), and 83% (312/376) for serum sodium quantile1 (Q1, 130–138.9 mmol/L), quantile 2 (Q2, 139–140.9 mmol/L), quantile 3 (Q3, 141–141.9 mmol/L), and quantile 4 (Q4, 142–155 mmol/L), respectively (*p* = 0.011). Multivariate odds ratio regression adjusted for risk factors demonstrates a 1-unit increment in the serum sodium raises the risk of sporadic Parkinson’s disease by 1.11 times, respectively. Smooth splines analysis suggested a linear association between levels of serum sodium and risk of sporadic Parkinson’s disease (P nonlinearity = 0.5). An interaction was observed between serum sodium and sex in their influence on sporadic Parkinson’s disease (*p* < 0.05). Further exploratory subgroup analysis within the age and BMI groups showed that there were no significant interactions between the subgroups (all *p* values for interaction were > 0.05). Additional sensitivity analyses supported the primary findings and indicated the conclusions are robust.

**Conclusion:**

This study highlights the influence of inappropriate serum sodium on the risk of incident sporadic Parkinson’s disease, independent of confounders. The link between serum sodium and sporadic Parkinson’s disease is linear.

## 1 Introduction

Parkinson’s disease (PD), in particular, is the second most prevalent neurodegenerative disorder after Alzheimer’s disease. It is hallmarked by the degeneration of dopaminergic neurons in the substantia nigra, a region of the midbrain (Obeso et al., 2010). This degeneration leads to a reduction in striatal dopamine, which is central to the characteristic motor symptoms of PD, including tremors, bradykinesia, rigidity, and postural instability (Coelho and Ferreira, 2012). The etiology of PD is multifactorial, involving genetic predispositions, environmental exposures, and cellular dysfunction. Recent research has highlighted the potential role of electrolyte imbalances in the pathophysiology of PD (Rajput et al., 2021).

The balance of electrolytes, including sodium, potassium, calcium, and magnesium, is essential for maintaining normal physiological functions (Lothian et al., 2013). Their distribution across cellular compartments is critical, and disruptions in these relationships can lead to neurological issues (Chen et al., 2016; Barnham and Bush, 2008). They play multiple roles in neurological health, influencing biological processes such as nerve impulse transmission, muscle contraction, and cellular signaling. Sodium is a key regulator of fluid balance, osmotic pressure, and nerve impulse transmission. It is well-established that alterations in sodium levels can significantly impact neurological function, with both hyponatremia and hypernatremia being associated with adverse outcomes in patients with PD (Mao et al., 2017). Elevated serum sodium was linked to increased AD pathology, as evidenced by higher amyloid PET uptake in susceptible brain regions such as the neocortex and limbic system (Chen et al., 2024). Studies have shown that changes in certain cognitive areas can already be seen in low-sodium individuals above the clinical threshold for hyponatremia (van der Burgh et al., 2023). This underscores the importance of serum sodium in the progression of AD. Ion channels in the dopaminergic neurons of the substantia nigra including epithelial sodium channels, G-protein-coupled inwardly rectifying potassium channel (GIRK), calcium-sensitive potassium channels, and N-methyl-D-aspartate (NMDA) receptors may be associated with the development of dyskinesia (Ravenscroft and Brotchie, 2000; Kobayashi and Ikeda, 2006; Dragicevic et al., 2015). The concentration of sodium is 11 millimolar (mm) both in gray and white matter in the brain, 10–14 mm within neuron cells, and 140 mm outside neuron cells (Madelin et al., 2014). A constant gradient of concentrations across the membrane plays a very important role in calcium regulation homeostasis, cell volume control, glucose transport, the electric potential of membranes, pH regulation, and neurotransmission. Disruptions in sodium balance can lead to altered neuronal excitability, which may be particularly detrimental in diseases like PD where neuronal integrity is already compromised (Ha et al., 2016).

Parkinson’s disease (PD) predominantly manifests as sporadic PD, which accounts for the majority of cases (Costa et al., 2022). In contrast, familial PD, which is caused by various genetic mutations, exhibits a wide range of clinical presentations due to the heterogeneity of the underlying genetic factors (Jia et al., 2022). To better understand the role of sodium in PD, we have chosen to focus our research on sporadic PD, as it represents the most common form of the disease and provides a more uniform clinical profile for investigation. This study aims to explore the relationship between sodium levels and sporadic PD, examining the potential for sodium imbalance to serve as a biomarker or therapeutic target. Understanding the intricate links between sodium homeostasis and sporadic PD may reveal novel avenues for therapeutic intervention and provide a deeper understanding of the complex pathophysiology underlying this debilitating disorder.

## 2 Methods

### 2.1 Participant selection and study grouping

**Figure 1:**
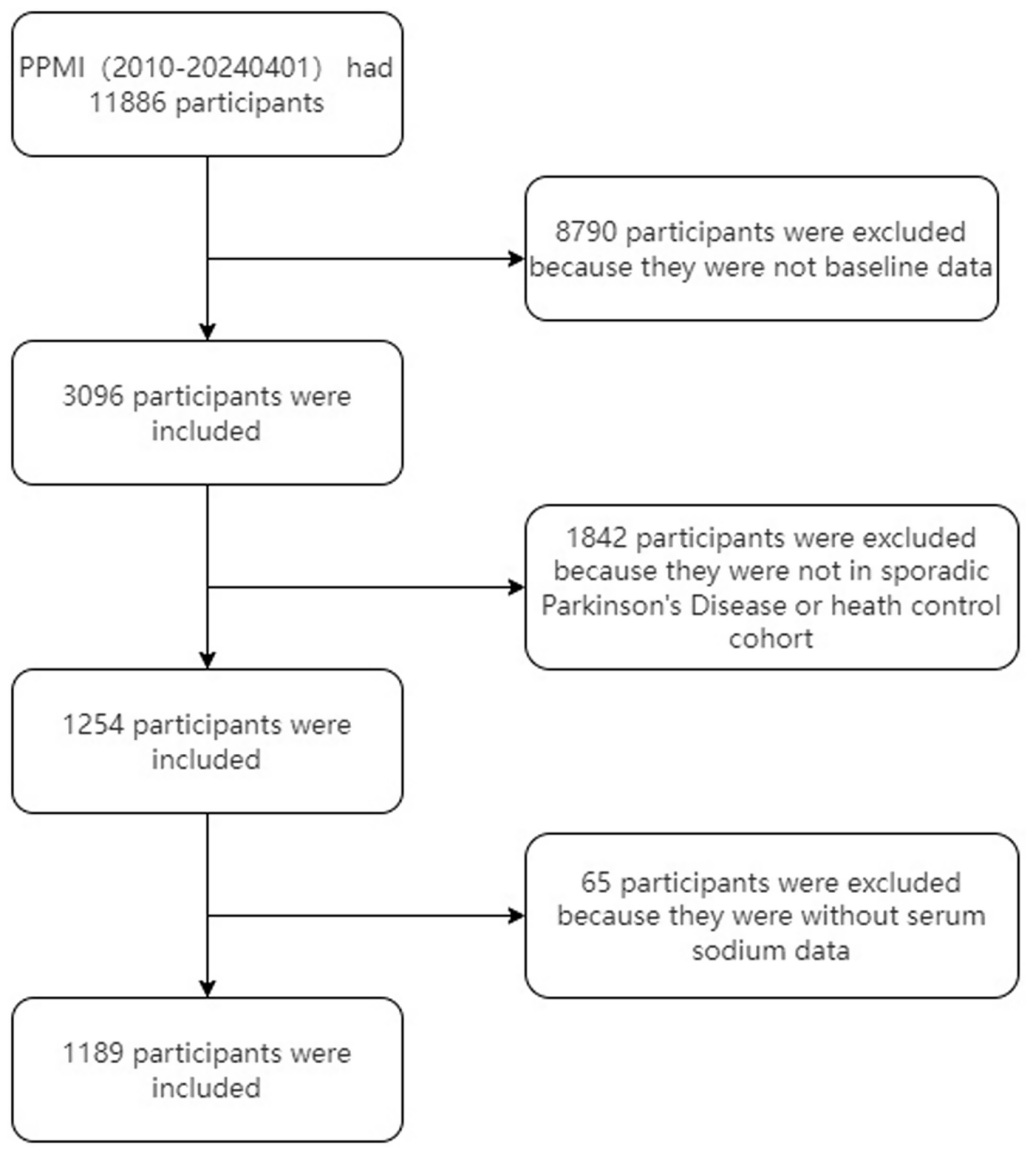
Flowchart of the study cohort.

Data were extracted from the PPMI database^1^ on January 4, 2024. The analysis was confined to baseline data collected at the time of participant enrollment. The flowchart for participant enrollment is presented in Figure 1.

The PPMI is a longitudinal, observational study designed to identify biomarkers of PD progression (Parkinson Progression Marker Initiative, 2011). Launched in 2010 by The Michael J. Fox Foundation, PPMI aims to improve the understanding of PD’s onset and progression by collecting comprehensive clinical, genetic, and biospecimen data from participants with PD, those at risk of developing PD, and healthy controls. The study has enrolled thousands of participants and has become a cornerstone of PD research, providing valuable insights into the disease’s natural history and potential therapeutic targets.

The PPMI studies are conducted in compliance with applicable regulations and ethical guidelines. Written informed consent is obtained from all participants, and the protocol has been granted approval by the local Institutional Review Boards of all participating institutions. All research activities adhere to the principles outlined in the 1964 Declaration of Helsinki and its subsequent revisions.

The inclusion criteria for sporadic Parkinson’s disease (PD) patients are as follows: individuals aged ≥30 years at the screening visit, with a PD diagnosis for ≤2 years; not expected to require PD medication within at least 6 months from baseline; presence of at least two of the following motor features: resting tremor, bradykinesia, rigidity (with either resting tremor or bradykinesia required), or asymmetric resting tremor/bradykinesia; Hoehn and Yahr stage I or II at baseline; willingness and medical ability to discontinue alpha-methyldopa, methylphenidate, amphetamine derivatives, or modafinil for at least 5 half-lives before SPECT imaging; eligibility confirmed by screening SPECT imaging; ability to provide informed consent; and, for females of childbearing potential, a negative pregnancy test on the day of screening SPECT imaging, with no pregnancy or lactation planned during the study. Orthostatic hypotension is diagnosed based on a change in systolic blood pressure of ≥20 mmHg or a change in diastolic blood pressure of ≥10 mmHg upon standing or during head-up tilt testing.

Biochemical analyses were conducted uniformly at Covance laboratories, as per the study protocol. These analyses included measurements of white blood cell (WBC), red blood cell (RBC), hemoglobin, lymphocytes, neutrophils, platelets, aspartate aminotransferase (AST), alanine aminotransferase (ALT), total protein, serum glucose, serum potassium, calcium, creatinine, serum uric acid, urea nitrogen, and albumin.

### 2.2 Statistical methods

To assess the normality of variable distributions, we employed histogram analysis, Q-Q plots, and the Kolmogorov–Smirnov test. Continuous variables that were normally distributed are reported as mean ± standard deviation (SD), while those with a skewed distribution are depicted as median [interquartile range (IQR)]. Categorical data are expressed as frequencies with percentages. Statistical comparisons across serum sodium groups were conducted using chi-square or Fisher’s exact tests for categorical variables, one-way ANOVA for variables with a normal distribution, and the Kruskal-Wallis H test for those with a skewed distribution.

We used multiple imputation, based on 5 replications and a chained equation approach method in the R mice procedure (van Buuren and Groothuis-Oudshoorn, 2011) to maximize statistical power and minimize bias that might occur account for missing data. The impact of serum sodium on sporadic PD was assessed by employing binary logistic regression models, presenting the results as odds ratios (OR) along with their corresponding 95% confidence intervals (CI). The models were adjusted for key covariates. Serum sodium was introduced into the models as a categorical variable divided into 4 quantiles. The selection of these confounders was based on clinical relevance, existing scientific literature (Kuwabara et al., 2020; Huang et al., 2022; Shimizu et al., 2021; van der Burgh et al., 2023; Zhou et al., 2024), all significant covariates identified in the univariate analysis, or their known associations with the outcomes of interest, including cases where they led to a change in the effect estimate exceeding 10%. A total of three models were constructed for analysis. Model 1 was unadjusted. Subsequently, Model 2 was further adjusted for age, sex, years of education, race, and body mass index, and orthostatic hypotension. Then, in Model 3, additional adjustments were made for lymphocytes, neutrophils, AST, creatinine, and serum uric acid.

We converted serum sodium into a categorical variable according to the quartile, and calculated the P for trend in order to verify the results of serum sodium as the continuous variable, and to examine the possibility of nonlinearity. We conducted restricted cubic spline model to develop smooth curves to examine the possible nonlinear dose–response associations between serum sodium and sporadic PD. In this model, serum sodium was used as a continuous variable with four knots (5th, 35th, 65th and 95th) suggested by Harrell et al. (1988). Non-linearity was assessed using a likelihood ratio test, comparing a model with linear terms only against one that included both linear and cubic spline terms. According to the smoothing curve, we further developed a two-piecewise linear regression model to find out the threshold effect, with adjustment for potential confounders. Interaction and stratified analyses were conducted according to subgroup variables.

To ensure the robustness of our findings and address potential confounders that may influence serum sodium levels, we conducted a series of sensitivity analyses. First, we excluded patients receiving diuretics or sodium-containing medications, given the potential impact of these drugs on serum sodium levels. This allowed us to isolate the effect of the drug of interest. Second, we excluded patients with renal insufficiency, defined as female creatinine levels >106 μmol/L and male creatinine levels >97 μmol/L, to ensure that our results were not confounded by impaired renal function. Third, we excluded patients with missing covariates to maintain the integrity of the analysis and avoid biases associated with incomplete data. Fourth, we excluded patients receiving levodopa therapy for PD (Finlay et al., 1971), as levodopa can affect serum sodium levels and thus potentially confound our results. Fifth, we used data collected during the second year after enrollment to account for potential short-term variability in serum sodium levels and provide a more stable assessment. Finally, we employed matching analysis weighting to adjust for potential confounding variables and ensure balanced groups, incorporating the following variables into the model: age, sex, BMI, race, education years, lymphocyte count, neutrophil count, AST, creatinine, serum uric acid, and orthostatic hypotension.

All analyses were performed using R Statistical Software (Version 4.2.2, http://www.R-project.org, The R Foundation) and Free Statistics analysis platform (Version 1.9, Beijing, China, http://www.clinicalscientists.cn/freestatistics). Free Statistics is a software package provides intuitive interfaces for most common analyses and data visualization. It uses R as the underlying statistical engine, and the graphical user interface (GUI) is written in Python. Most analyses can be done with just a few clicks. It is designed for reproducible analysis and interactive computing. A two-sided *p* value <0.05 was considered statistically significant.

## 3 Result

### 3.1 Population characterization

We included 1,189 patients aged 63 ± 9.9 years, white was 93.5%, and male were 64.3%. Of these, the overall prevalence of sporadic Parkinson’s disease was 77.5%. The baseline characteristics of the groups stratified by serum sodium are shown in Table 1. The four groups differed in age, monocytes, eosinophils, RBC, platelets, total protein, serum chloride and calcium (all *p* value <0.05). Otherwise, the distribution of patients’ characteristics (sex, education years, race, body mass index, WBC, lymphocytes, neutrophils, hemoglobin, MCV, ALT, AST, albumin, creatinine, serum uric acid, urea nitrogen, serum glucose, serum potassium) between serum sodium groups was similar (all *p* value >0.05).

**Table 1:** Clinical characteristics of the study population by serum sodium levels.

Variables	Total (*n* = 1,189)	Q1 (*n* = 199)	Q2 (*n* = 391)	Q3 (*n* = 223)	Q4 (*n* = 376)	*p*
Cohort, *n* (%)						0.011
Health Control	268 (22.5)	56 (28.1)	96 (24.6)	52 (23.3)	64 (17)	
Sporadic PD	921 (77.5)	143 (71.9)	295 (75.4)	171 (76.7)	312 (83)	
Age, Mean ± SD	63.0 ± 9.9	64.1 ± 10.6	62.0 ± 9.5	62.8 ± 10.4	63.7 ± 9.4	0.033
Sex, *n* (%)						0.493
Female	424 (35.7)	62 (31.2)	139 (35.5)	83 (37.2)	140 (37.2)	
Male	765 (64.3)	137 (68.8)	252 (64.5)	140 (62.8)	236 (62.8)	
Education years, Mean ± SD	16.0 ± 3.1	15.9 ± 3.3	16.0 ± 3.1	15.9 ± 3.0	16.2 ± 3.0	0.698
Race, *n* (%)						0.658
White	1,106 (93.5)	182 (92.4)	366 (94.3)	210 (94.6)	348 (92.6)	
Black	23 (1.9)	7 (3.6)	7 (1.8)	4 (1.8)	5 (1.3)	
Asian	18 (1.5)	3 (1.5)	4 (1)	2 (0.9)	9 (2.4)	
Other (includes multi-racial)	36 (3.0)	5 (2.5)	11 (2.8)	6 (2.7)	14 (3.7)	
BMI (kg/m^2^), Mean ± SD	26.3 (24.0, 29.6)	27.1 (24.0, 30.5)	26.3 (23.9, 29.3)	26.5 (24.2, 29.6)	25.9 (23.7, 28.9)	0.093
Mean caudate, Mean ± SD	2.2 ± 0.7	2.3 ± 0.8	2.2 ± 0.8	2.3 ± 0.7	2.2 ± 0.7	0.039
Mean striatum, Mean ± SD	1.7 ± 0.7	1.8 ± 0.7	1.7 ± 0.7	1.7 ± 0.6	1.6 ± 0.6	0.018
Mean putamen, Median (IQR)	1.2 ± 0.7	1.3 ± 0.7	1.2 ± 0.7	1.2 ± 0.6	1.1 ± 0.6	0.014
WBC (x10^3^/μL), Mean ± SD	6.0 ± 1.6	6.1 ± 1.8	6.1 ± 1.7	6.0 ± 1.5	5.9 ± 1.6	0.403
Lymphocytes (x10^3^/μL), Mean ± SD	1.6 ± 0.6	1.7 ± 0.8	1.6 ± 0.5	1.6 ± 0.5	1.6 ± 0.7	0.819
Monocytes (x10^3^/μL), Mean ± SD	0.38 ± 0.14	0.42 ± 0.16	0.39 ± 0.14	0.38 ± 0.12	0.37 ± 0.12	< 0.001
Neutrophils (x10^3^/μL), Mean ± SD	3.8 ± 1.3	3.8 ± 1.3	3.9 ± 1.4	3.8 ± 1.2	3.7 ± 1.2	0.389
RBC (x10^6^/μL), Mean ± SD	4.66 ± 0.43	4.57 ± 0.40	4.70 ± 0.43	4.69 ± 0.44	4.65 ± 0.42	0.004
Hemoglobin (g/L), Mean ± SD	142.3 ± 12.5	140.0 ± 11.7	142.8 ± 12.6	142.3 ± 12.9	142.7 ± 12.4	0.054
Platelets (x10^3^/μL), Mean ± SD	244.8 ± 58.7	252.8 ± 63.2	245.1 ± 59.1	247.7 ± 54.6	238.8 ± 57.8	0.049
AST (U/L), Median (IQR)	22.9 ± 11.4	24.4 ± 17.9	22.5 ± 12.5	22.6 ± 6.6	22.6 ± 7.0	0.2
ALT (U/L), Median (IQR)	19.0 (15.0, 25.0)	20.0 (16.0, 26.0)	19.0 (15.0, 24.0)	19.0 (15.0, 25.0)	20.0 (16.0, 27.0)	0.216
Total Protein (g/L), Mean ± SD	69.6 ± 3.9	70.0 ± 4.6	69.9 ± 3.8	69.8 ± 3.9	69.0 ± 3.7	0.002
Albumin (g/L), Mean ± SD	43.4 ± 3.7	43.5 ± 3.9	43.6 ± 3.6	43.2 ± 4.1	43.3 ± 3.6	0.594
Creatinine (umol/L), Mean ± SD	83.3 ± 17.6	82.2 ± 16.1	82.8 ± 20.0	84.1 ± 17.0	83.8 ± 16.0	0.621
Serum Uric Acid (umol/L), Mean ± SD	311.7 ± 77.2	309.7 ± 82.4	316.2 ± 79.2	309.9 ± 77.8	309.1 ± 71.7	0.569
Urea Nitrogen (mmol/L), Mean ± SD	6.0 ± 1.6	5.9 ± 1.7	6.0 ± 1.6	5.9 ± 1.5	6.0 ± 1.6	0.974
Serum Glucose (mmol/L), Mean ± SD	5.6 ± 1.1	5.7 ± 1.4	5.6 ± 0.9	5.6 ± 1.2	5.6 ± 1.0	0.421
Serum Potassium (mmol/L), Mean ± SD	4.4 ± 0.4	4.4 ± 0.4	4.4 ± 0.4	4.3 ± 0.3	4.4 ± 0.4	0.597
Serum Sodium (mmol/L), Mean ± SD	140.5 ± 2.3	136.9 ± 1.6	139.6 ± 0.5	141.0 ± 0.0	142.9 ± 1.3	< 0.001
Calcium (mmol/L), Mean ± SD	2.40 ± 0.10	2.38 ± 0.10	2.41 ± 0.11	2.40 ± 0.09	2.40 ± 0.09	0.002
Orthostatic hypotension, *n* (%)						0.243
No	1,029 (86.9)	176 (88.4)	346 (88.9)	191 (86.4)	316 (84.3)	
Yes	155 (13.1)	23 (11.6)	43 (11.1)	30 (13.6)	59 (15.7)	

N, number; Q1: 130–138.9 mmol/L; Q2: 139–140.9 mmol/L; Q3:141–141.9 mmol/L; Q4:142–155 mmol/L; PD, Parkinson’s disease; BMI, body mass index; RBC, red blood cell; WBC, white blood cell; AST, Aspartate Aminotransferase; race other, includes multi-racial; Numbers that do not add up to 100% are attributable to missing data.

### 3.2 Multivariate analysis of serum sodium and related factors of sporadic PD

Table 2 delineates the findings from the multivariate logistic regression analysis, which probed the relationship between serum sodium levels and the risk of sporadic Parkinson’s disease. Elevated serum sodium levels were correlated with a heightened risk of PPD, with an odds ratio (OR) of 1.11 per unit increase (95% confidence interval [CI], 1.04–1.18; *p* = 0.001), after accounting for confounding variables (Table 2, multivariate analysis). Within the multivariate logistic regression framework, when compared to the reference group with the lowest serum sodium levels (Q1: 130–138.9 mmol/L), the adjusted ORs for sporadic PD across increasing quartiles of serum sodium were as follows: Q2 (139–140.9 mmol/L) yielded an OR of 1.22 (95% CI, 0.82–1.83; *p* = 0.324), Q3 (141–141.9 mmol/L) an OR of 1.3 (95% CI, 0.83–2.05; *p* = 0.258), and Q4 (142–155 mmol/L) an OR of 1.94 (95% CI, 1.27–2.98; *p* = 0.002), respectively.

**Table 2:** Results of univariate and multivariate logistic regression analyses of associations between serum sodium and sporadic Parkinson’s disease.

Variable	*n* total	Model 1		Model 2		Mode 3	
		OR (95%CI)	*P* value	OR (95%CI)	*P* value	OR (95%CI)	*P* value
Serum sodium	1,189	1.1 (1.04 ~ 1.16)	0.002	1.1 (1.03 ~ 1.16)	0.002	1.11 (1.04 ~ 1.18)	0.001
Quartiles							
Q1	199	1(Ref)		1(Ref)		1(Ref)	
Q2	391	1.2 (0.82 ~ 1.77)	0.346	1.21 (0.82 ~ 1.8)	0.331	1.22 (0.82 ~ 1.83)	0.324
Q3	223	1.29 (0.83 ~ 2)	0.258	1.28 (0.82 ~ 2)	0.278	1.3 (0.83 ~ 2.05)	0.258
Q4	376	1.91 (1.27 ~ 2.88)	0.002	1.88 (1.24 ~ 2.86)	0.003	1.94 (1.27 ~ 2.98)	0.002
p for trend	1,189		0.001		0.002		0.001

Q1: 130–138.9 mmol/L; Q2: 139–140.9 mmol/L; Q3:141–141.9 mmol/L; Q4:142–155 mmol/L; PD, Parkinson’s disease; n, number. Model 1: unadjusted. Model 2: Adjusted factors include age, sex, education years, race, body mass index, and orthostatic hypotension. Model 3: Adjusted Model 2, lymphocytes, neutrophils, AST, creatinine, and serum uric acid.

**Figure 2:**
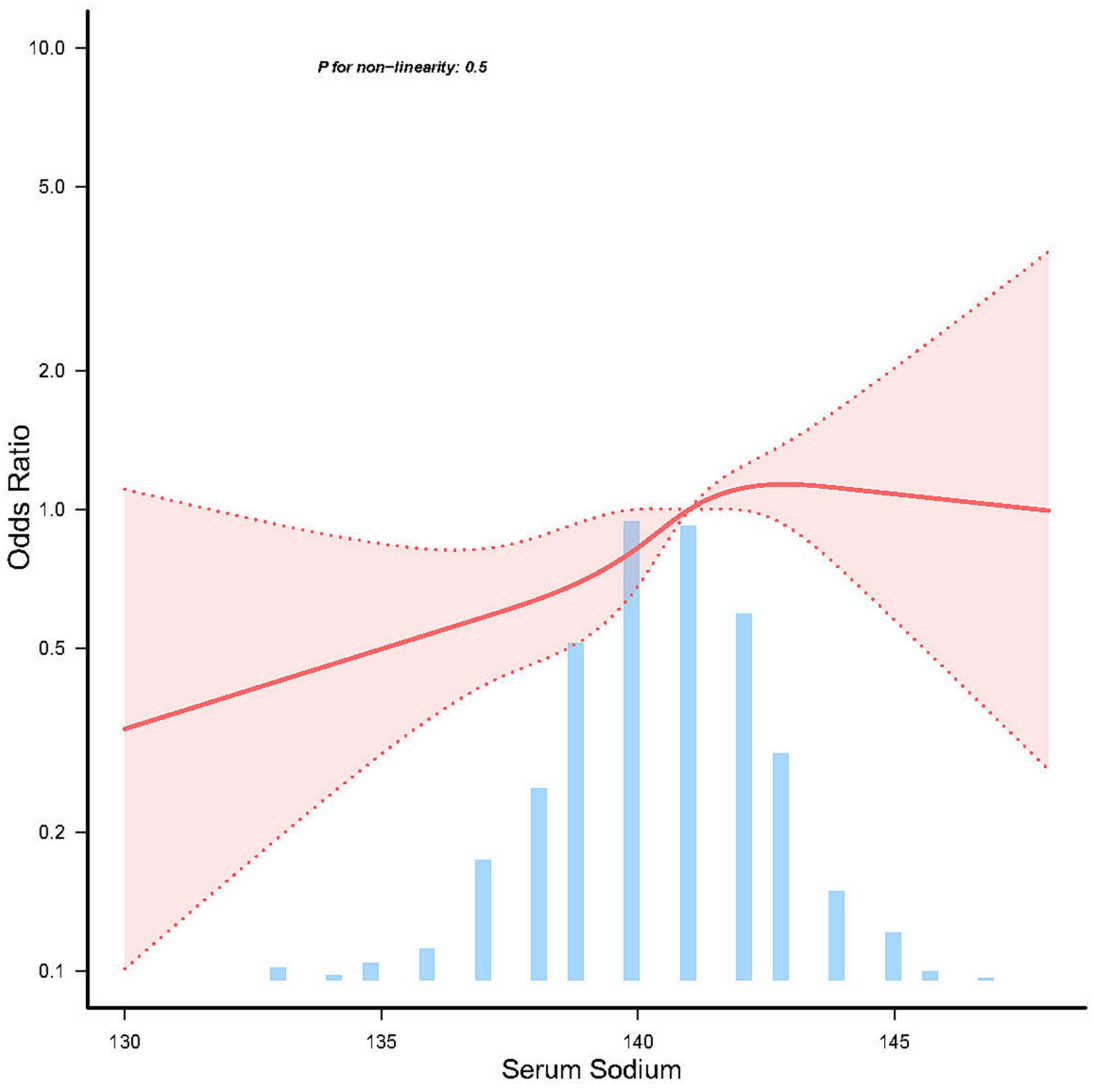
Linear dose response relationship between serum sodium and sporadic Parkinson’s disease. Adjustment factors included age, sex, education years, race, body mass index, lymphocytes, neutrophils, AST, creatinine, serum uric acid, and orthostatic hypotension. The red line and red area represent the estimated values and their corresponding 95% confidence intervals, respectively.

The relationship between serum sodium and the risk of sporadic PD, as depicted in the restricted cubic spline (RCS) model, revealed a linear association (*p* = 0.5) (Figure 2).

### 3.3 Subgroup analysis and sensitivity analysis

**Figure 3:**
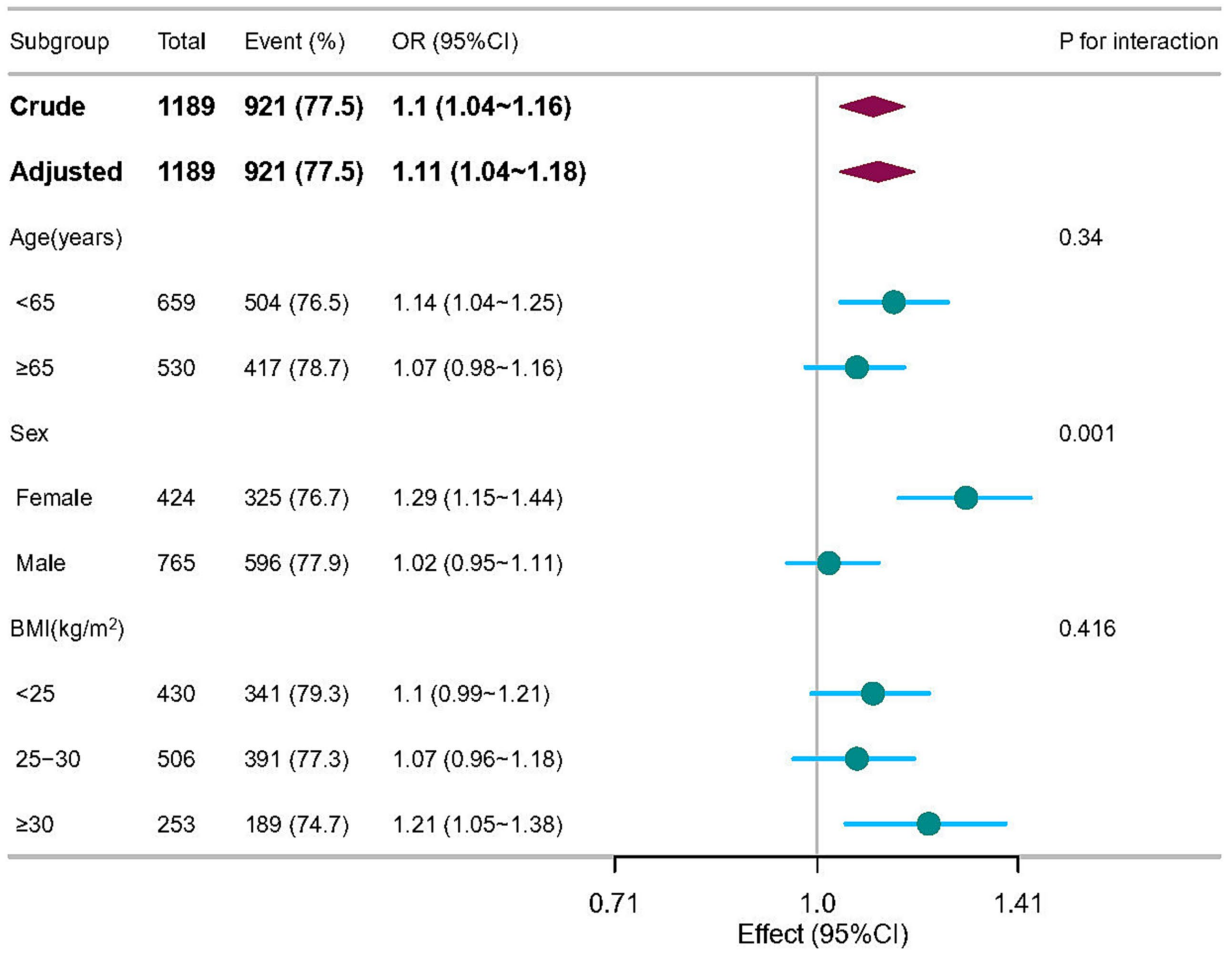
Subgroup analyses of the associations between serum sodium and sporadic Parkinson’s disease adjusted for age, sex, education years, race, body mass index, lymphocytes, neutrophils, AST, creatinine, serum uric acid, and orthostatic hypotension. In each case, the model was not adjusted for the stratification variable.

Additional subgroup and sensitivity analyses concerning the role of age, sex and BMI, are presented and shown in the Figure 3. As expected, in stratified analysis by sex, a greater benefit of serum sodium was observed for increasing the risk of sporadic PD among participants with female group (OR = 1.29, 95% CI = 1.15–1.44) compared to those with male group at baseline (OR = 1.02, 95% CI = 0.95–1.11; P interaction <0.05).

Table 3 summarizes the results of sensitivity analyses. After excluding participants treated with diuretics or sodium-containing medications, serum sodium was associated with sporadic PD (OR, 1.11; 95% CI, 1.04–1.19). After excluding patients with renal insufficiency, the OR was 1.12 (95% CI, 1.05–1.19). After excluding patients with missing covariates, the OR was 1.1 (95% CI, 1.03–1.17). After excluding patients receiving levodopa therapy for PD, the OR was 1.11 (95% CI, 1.04–1.18). In addition, used data collected during the second year after enrollment, the association between serum sodium and sporadic PD remained (OR, 1.09; 95% CI, 1.01–1.19). After inverse probability treatment weighted analyses, the OR was 1.42 (95% CI, 1.08–1.87).

**Table 3:** Results of multivariate logistic regression analyses of associations between serum sodium and sporadic PD in sensitivity analyses.

Analysis	Variable	*n* total	Model 1		Model 2		Model 3	
			OR (95%CI)	*P* value	OR (95%CI)	*P* value	OR (95%CI)	*P* value
Exclusion of patients receiving diuretics or sodium-containing medications	Serum sodium	937	1.09 (1.02 ~ 1.16)	0.009	1.09 (1.02 ~ 1.17)	0.009	1.11 (1.04 ~ 1.19)	0.002
Exclusion of patients with renal insufficiency	Serum sodium	1,107	1.11 (1.04 ~ 1.18)	0.001	1.11 (1.04 ~ 1.18)	0.001	1.12 (1.05 ~ 1.19)	0.001
Exclusion of patients with missing covariates	Serum sodium	1,108	1.09 (1.03 ~ 1.16)	0.003	1.09 (1.03 ~ 1.16)	0.004	1.1 (1.03 ~ 1.17)	0.003
Exclusion of patients receiving levodopa therapy	Serum sodium	1,178	1.1 (1.04 ~ 1.16)	0.002	1.1 (1.03 ~ 1.16)	0.002	1.11 (1.04 ~ 1.18)	0.001
Use of data from the second year of follow-up after enrollment	Serum sodium	628	1.09 (1.01 ~ 1.18)	0.037	1.08 (1 ~ 1.17)	0.047	1.09 (1.01 ~ 1.19)	0.034
Inverse probability treatment weighted analyses	Serum sodium	1,189	/	/	/	/	1.42 (1.08 ~ 1.87)	0.012

PD, Parkinson’s disease; n, number. Model 1: unadjusted. Model 2: Adjusted factors include age, sex, education years, race, body mass index, and orthostatic hypotension. Model 3: Adjusted Model2, lymphocytes, neutrophils, AST, creatinine, and serum uric acid.

## 4 Discussion

In this retrospective cross-sectional investigation, we initially established that serum sodium levels were significantly and independently linked to a 12% heightened risk of sporadic PD. Results were consistent across clinical subgroups and in sensitivity analyses. These observations would have important implications for current sporadic PD. These findings carry significant implications for the management of sporadic PD, particularly in the white population, suggesting that sodium homeostasis may play a crucial role in disease progression and should be considered in therapeutic strategies.

Previous research has yielded mixed findings regarding the association between serum sodium and PD. One study has indicated a slight elevation in serum sodium levels in the PD group compared to the healthy control group (139.6 mmol/L vs. 139.5 mmol/L, respectively). However, this difference was minimal and not statistically significant (*p* > 0.05), suggesting that serum sodium levels are not meaningfully different between the two groups (Zhou et al., 2024). This may be attributed to the overall lower serum sodium levels in that study, with the PD group having a mean serum sodium level of 139 ± 3.0 mmol/L. In contrast, our study observed a higher mean serum sodium level of 140.6 ± 2.3 mmol/L in sporadic PD group, which allows us to explore the relationship between higher serum sodium levels and PD more effectively (Table 4). For instance, in our study, when comparing the second (Q2) or third quartile (Q3) to the first quartile (Q1), the differences were not statistically significant (*p* > 0.05). However, in the highest quartile group (Q4), which represents the highest serum sodium levels, a statistically significant association with PD was observed (*p* < 0.05). This discrepancy may provide novel insights into the role of sodium in the sporadic PD. Other studies have reported an inverse correlation between serum sodium levels and movement disorders in PD, with a corresponding odds ratio of 0.783 (95% CI, 0.642–0.955) (Mao et al., 2017). This inverse relationship may be attributed to the fact that levodopa, a common treatment for PD, was known to increase the excretion of sodium and potassium (Finlay et al., 1971). Consequently, patients with more severe dyskinesia, who typically require higher doses of levodopa (Mao et al., 2017), may exhibit lower serum sodium levels. It is important to note that the diagnosis of PD in this study was based on a questionnaire assessing whether participants had received treatment for PD. However, only 11 of the sporadic PD patients included in our study received PD-related drugs, which may be the reason why this study is inconsistent with our findings.

**Table 4:** Clinical characteristics of the study population by sporadic PD.

Variables	Total (*n* = 1,189)	Sporadic PD		*p*
		Yes (*n* = 921)	No (*n* = 268)	
Serum Sodium, Mean ± SD	140.5 ± 2.3	140.6 ± 2.3	140.1 ± 2.4	0.002
Age, Mean ± SD	63.0 ± 9.9	63.4 ± 9.3	62.0 ± 11.5	0.046
Sex, *n* (%)				0.619
Female	424 (35.7)	325 (35.3)	99 (36.9)	
Male	765 (64.3)	596 (64.7)	169 (63.1)	
Education years, Mean ± SD	16.0 ± 3.1	16.0 ± 3.1	16.1 ± 3.1	0.455
Race, *n* (%)				0.015
White	1,106 (93.5)	864 (94.3)	242 (90.6)	
Black	23 (1.9)	11 (1.2)	12 (4.5)	
Asian	18 (1.5)	13 (1.4)	5 (1.9)	
Other (includes multi-racial)	36 (3.0)	28 (3.1)	8 (3)	
BMI (kg/m^2^), Mean ± SD	74.7 ± 1637.3	88.7 ± 1862.1	27.0 ± 4.5	0.588
Mean caudate, Mean ± SD	2.2 ± 0.7	2.0 ± 0.6	3.0 ± 0.6	< 0.001
Mean striatum, Mean ± SD	1.7 ± 0.7	1.4 ± 0.4	2.6 ± 0.6	< 0.001
Mean putamen, Mean ± SD	1.2 ± 0.7	0.9 ± 0.3	2.2 ± 0.6	< 0.001
WBC (x10^3^/μL), Mean ± SD	6.0 ± 1.6	6.0 ± 1.6	6.2 ± 1.8	0.158
Lymphocytes (x10^3^/μL), Mean ± SD	1.6 ± 0.6	1.6 ± 0.6	1.8 ± 0.8	< 0.001
Monocytes (x10^3^/μL), Mean ± SD	0.38 ± 0.14	0.38 ± 0.13	0.40 ± 0.15	0.008
Neutrophils (x10^3^/μL), Mean ± SD	3.8 ± 1.3	3.8 ± 1.3	3.7 ± 1.3	0.264
RBC (x10^6/μL), Mean ± SD	4.66 ± 0.43	4.66 ± 0.43	4.66 ± 0.40	0.982
Hemoglobin (g/L), Mean ± SD	142.3 ± 12.5	142.3 ± 12.6	142.0 ± 12.2	0.741
Platelets (x10^3^/μL), Mean ± SD	244.8 ± 58.7	242.8 ± 57.3	251.9 ± 63.0	0.03
AST (U/L), Mean ± SD	22.9 ± 11.4	22.3 ± 9.7	24.9 ± 15.6	< 0.001
ALT (U/L), Median (IQR)	19.0 (15.0, 25.0)	19.0 (15.0, 25.0)	20.0 (16.0, 27.0)	0.059
Total Protein (g/L), Mean ± SD	69.6 ± 3.9	69.6 ± 4.0	69.7 ± 3.9	0.843
Albumin (g/L), Mean ± SD	43.4 ± 3.7	43.9 ± 3.7	41.9 ± 3.5	< 0.001
Creatinine (umol/L), Mean ± SD	83.3 ± 17.6	83.0 ± 17.7	84.1 ± 17.2	0.389
Serum Uric Acid (umol/L), Mean ± SD	311.7 ± 77.2	309.1 ± 76.1	320.8 ± 80.2	0.029
Urea Nitrogen (mmol/L), Mean ± SD	6.0 ± 1.6	6.0 ± 1.6	6.0 ± 1.6	0.936
Serum Glucose (mmol/L), Mean ± SD	5.6 ± 1.1	5.6 ± 1.1	5.5 ± 1.0	0.487
Serum Potassium (mmol/L), Mean ± SD	4.4 ± 0.4	4.4 ± 0.3	4.3 ± 0.4	0.367
Calcium (mmol/L), Mean ± SD	2.40 ± 0.10	2.40 ± 0.10	2.40 ± 0.10	0.789
Orthostatic hypotension, *n* (%)				0.038
No	1,029 (86.9)	786 (85.8)	243 (90.7)	
Yes	155 (13.1)	130 (14.2)	25 (9.3)	

Sodium channels may play a pivotal role in the pathophysiology of PD. Studies have shown that in PD models, the expression of Nav1.1, Nav1.3, and Nav1.6 in the hippocampus increases after dopamine depletion (Wang et al., 2019). The use of sodium channel blockers, such as phenytoin, can significantly improve cognitive impairments, suggesting that abnormal activation of sodium channels may be related to the pathological mechanisms of PD. Additionally, the expression of Nav1.1 in GABAergic interneurons of the external segment of the globus pallidus increases (Liu et al., 2021), which may enhance inhibitory neural activity to compensate for the abnormal neural activity caused by PD (Saunders et al., 2016). Nav1.3 may also be re-expressed during PD progression as a compensatory mechanism in response to the degeneration of dopaminergic neurons (Wang et al., 2019). Mitochondrial dysfunction leads to calcium imbalance, which activates the mitochondrial permeability transition pore (MPTP) and triggers apoptosis. The abnormal exchange of sodium ions further disrupts the ionic balance across the mitochondrial membrane, worsening oxidative stress. Additionally, environmental toxins such as MPTP (Ramsay et al., 1986) and rotenone (Betarbet et al., 2000) inhibit mitochondrial complex I, leading to decreased ATP production and increased reactive oxygen species (ROS). The dysfunction of NCX may further exacerbate this oxidative stress (Surmeier et al., 2011), thereby promoting the pathological progression of PD. Utilizing Ultra-High Field (7 T) MRI, an elevation in sodium concentration within the substantia nigra has been identified in the early stages of PD (Grimaldi et al., 2021). The accumulation of sodium may affect the activity of other structures within the SN-basal ganglia-thalamic-cortical circuit, potentially impacting the generation of action potentials in dopaminergic neurons and the function of GABAergic inhibition in the basal ganglia (Lozovaya et al., 2018). These insights underscore the significance of sodium homeostasis in the context of PD and warrant further exploration into its role in the pathogenesis of PD. In this paper, the correlation between serum sodium level and sporadic PD is clear, but the specific causal relationship and the pathological mechanism of how sodium participates in sporadic PD still need to be further studied.

Abnormal serum sodium levels may influence the risk of PD through multiple mechanisms. Firstly, changes in serum sodium concentration can disrupt cellular osmotic balance, thereby impairing normal neuronal function. Specifically, in dopaminergic neurons, the activity of the sodium-potassium ATPase (Na+/K + -ATPase) is critical for maintaining intracellular and extracellular ion balance. Abnormal sodium levels may interfere with this process, leading to neuronal dysfunction (Sterns, 2015). Secondly, dopamine autoxidation is a significant factor in the pathogenesis of PD, and the stability of dopamine is highly dependent on an acidic environment. Dopamine is primarily stored in acidic vesicles, where the low pH environment prevents its autoxidation (Umek et al., 2018). The vesicular monoamine transporter 2 (VMAT2) is responsible for transporting dopamine into these vesicles, and its activity relies on the proton gradient maintained by V-ATPase (Kosmidis et al., 2022). Therefore, any factors affecting V-ATPase activity (such as changes in sodium concentration) may indirectly impact dopamine storage and stability, thereby increasing the risk of PD. Additionally, studies have shown that chronic hyponatremia is associated with an increased risk of osteoporosis and fractures (Sterns, 2015), and osteoporosis itself is considered a potential risk factor for PD (Feng et al., 2021). In summary, serum sodium levels may contribute to the risk of PD by affecting ion balance in dopaminergic neurons, dopamine stability, and bone metabolism.

This manuscript presents several methodological strengths. Initially, our investigation of the association between serum sodium levels and sporadic PD employed multivariate logistic regression models, which effectively controlled for potential confounders, thereby reducing bias. Additionally, we conducted smooth curve fitting to delineate the linear relationship between serum sodium and sporadic PD. Furthermore, the robustness of our findings was confirmed through stratified subgroup and sensitivity analyses, which explored the serum sodium-sporadic PD relationship across diverse populations.

However, this study is not without its limitations. Firstly, the observational nature of our research limits direct comparability to the gold standard of randomized controlled trials (RCTs). Secondly, the cross-sectional design precludes a comprehensive understanding of the causal mechanisms underlying the observed associations. Thirdly, our study establishes a correlation rather than a causal link between serum sodium and sporadic PD, underscoring the necessity for future prospective cohort studies to affirm our observations. Fourthly, even though regression models were constructed, and stratified analyses and sensitivity analysis were performed, residual confounding effects from unmeasured or unknown factors could not be entirely excluded. Finally, the retrospective nature of the study and the uneven sample size distribution may introduce biases inherent to this design. Specifically, the interpretation of odds ratios (ORs) as estimates of relative risk should be approached with caution, particularly given the relatively high prevalence of the outcome in our study population. In such scenarios, ORs are known to overestimate RRs, which may lead to an overinterpretation of the strength of associations observed. Despite these limitations, our study provides valuable insights into the relationship between serum sodium levels and sporadic PD, highlighting the need for further investigation in a prospective setting with more balanced sample sizes. The findings should be interpreted within the context of these methodological constraints, and future research should aim to address these limitations through more robust study designs.

## 5 Conclusion

This study underscores the impact of serum sodium levels on the risk of sporadic PD, independent of other confounding variables. Our findings reveal a linear relationship between serum sodium and sporadic PD. These insights are of significant interest and may hold crucial implications for understanding the pathogenesis of PD and the development of disease-modifying therapies. Further validation and confirmation of our results are warranted to solidify these findings.

## Data Availability

Publicly available datasets were analyzed in this study. This data can be found at: http://www.ppmi-info.org (assessed on April 1, 2024).

## Footnotes

^1^ http://www.ppmi-info.org
